# A Finite Element Simulation-Informed Machine Learning Framework for Screening Average Thermal Stress Responses in SLM-Fabricated 316L Stainless Steel

**DOI:** 10.3390/ma19102088

**Published:** 2026-05-15

**Authors:** Yuan Zheng, Shaoding Sheng

**Affiliations:** Department of Materials Science and Engineering, Anhui University of Science and Technology, Huainan 232001, China; qingflowers@163.com

**Keywords:** selective laser melting, 316L stainless steel, machine learning surrogate model, comparative thermal stress indicator, residual stress, finite element simulation

## Abstract

**Highlights:**

- Among the nine evaluated regression models, BPNN achieved the best predictive performance for the finite-element-generated *σ*_avg_ dataset, and BPNN was selected as the representative surrogate model because it combines high predictive accuracy with direct applicability to the present structured tabular dataset.
- Within the investigated simulation window, laser power was identified as the dominant variable governing the *σ*_avg_-based response, whereas substrate preheating temperature showed a secondary mitigating effect, and scanning speed mainly influenced the response through coupled interactions.
- Within the investigated parameter window, conditions near 180–200 W were associated with a relatively lower predicted *σ*_avg_ level. Under the limited experimental conditions examined here, samples fabricated near 200 W also exhibited improved metallographic quality and lower XRD-derived macroscopic stress estimates.
- A finite element simulation-driven machine learning surrogate model can improve the efficiency of comparative process-window screening in SLM when large numbers of parameter combinations need to be ranked.

**Abstract:**

To improve the efficiency of comparative process-window screening in selective laser melting (SLM), this study developed a finite element simulation-driven machine learning framework for 316L stainless steel. A simulation dataset covering laser power (LP), scanning speed (SS), heat-source diameter (HSD), and substrate preheating temperature (SPH) was generated using ANSYS and used to train nine regression models. In the present work, the primary machine learning target was defined as the simulated average thermal stress, *σ*_avg_, which is used as a simulation-derived comparative thermal stress indicator for ranking process conditions within the investigated parameter window rather than as a direct prediction of the final residual-stress field. Among the evaluated models, the Backpropagation Neural Network (BPNN) showed the best predictive performance and was selected as the representative surrogate model because of its strong predictive accuracy, stable behavior, and direct applicability to the present structured tabular dataset. Shapley additive explanations (SHAP) and partial dependence plots (PDPs) indicated that LP is the dominant variable governing the *σ*_avg_-based response, followed by SPH, whereas SS and HSD mainly affect the response through secondary or coupled effects. Within the investigated parameter window, conditions near 180–200 W corresponded to a relatively lower predicted *σ*_avg_ level. Experimental observations provided limited but meaningful trend-level support for the simulation-guided screening results: metallographic examination showed improved forming quality near 200 W, while XRD-derived macroscopic stress estimates exhibited a similar variation trend to the simulated *σ*_avg_ values under the tested LP–SS conditions. These results suggest that the proposed framework can serve as an efficient surrogate-based tool for comparative parameter screening in SLM-fabricated 316L stainless steel within the assumptions and parameter range of the present model.

## 1. Introduction

In recent years, with the growing demand for complex microstructured materials in high-end manufacturing sectors such as electronics, healthcare, automotive, and biotechnology [1,2], traditional micro-manufacturing methods have increasingly exposed their limitations in precision, flexibility, and applicability [3,4]. Additive manufacturing (AM) technologies, particularly selective laser melting (SLM), have gradually become crucial manufacturing techniques in these fields due to their significant advantages in rapidly and accurately producing complex structural components [5,6]. SLM is now widely applied in industries like aerospace, military equipment, biomedical devices, and precision instruments [7,8,9]. However, SLM still faces numerous challenges in practical applications, particularly the issue of residual stress generated during the manufacturing process. Due to the high-energy laser beam’s localized heating and rapid cooling in a very short time, significant temperature gradients and thermal stresses form within the material, leading to considerable residual stress [10]. This residual stress not only directly impacts the dimensional accuracy and microstructure of the components but can also cause deformation and cracking, severely reducing the mechanical properties and reliability of the fabricated parts [11,12,13]. Consequently, accurately predicting and effectively controlling residual stress is critical for ensuring the quality of SLM manufacturing and optimizing the process.

Currently, residual-stress-related process assessment in SLM mainly relies on experiments [14,15] and numerical simulations [16]. Experimental measurements can provide direct information on the final stress state, but they are time-consuming, costly, and inefficient for high-dimensional parameter screening. Numerical simulations, especially transient thermo-mechanical finite element analysis [17,18], offer important physical insight into temperature and stress evolution during fabrication [19]. However, their computational cost remains prohibitive when a large number of parameter combinations must be evaluated. In parallel, machine learning has recently emerged as a promising tool for process optimization in additive manufacturing [20,21] because it can approximate complex process–structure–property relationships at a much lower computational cost [22,23]. Nevertheless, purely data-driven models often lack clear physical grounding, while high-fidelity simulations alone are difficult to use for rapid iterative optimization [24,25].

To address the trade-off between simulation fidelity and screening efficiency, the present study proposes a finite element simulation-driven machine learning framework for SLM-fabricated 316L stainless steel. In this framework, the simulated average thermal stress, *σ*_avg_, is adopted as the primary machine learning target for comparative process screening within the investigated parameter space. It should be emphasized that *σ*_avg_ is used here as a simulation-derived comparative thermal stress indicator for ranking parameter combinations under the assumptions of the adopted model, rather than as a direct prediction of the full final residual-stress field of fabricated components. Compared with local instantaneous peak-stress descriptors, *σ*_avg_ provides a more stable global response quantity within the present simulation framework and is therefore more suitable for subsequent trend-level comparison with post-process macroscopic stress estimates. ANSYS simulations were used to generate a dataset covering different combinations of laser power, scanning speed, heat-source diameter, and substrate preheating temperature. Based on this finite-element-generated dataset, nine regression models were trained and compared to identify a suitable surrogate model for rapid comparative screening. The BPNN showed the best predictive performance and was selected as the representative model for further interpretation because it provides strong predictive accuracy and is more directly applicable to the present structured static tabular dataset. Finally, metallographic observations and XRD-based macroscopic stress estimates were used to provide limited experimental support for the dominant trends identified by the simulation-guided screening framework. Through this combined approach, the present study aims to establish an efficient and interpretable route for *σ*_avg_-oriented comparative parameter screening and local process-window identification in SLM-fabricated 316L stainless steel.

## 2. Materials and Methods

### 2.1. Material

The metal powder used in both the numerical simulations and experimental studies was 316L stainless steel, and its key material parameters are summarized in Table 1. Specifically, the 316L stainless steel powder used in the experimental study was a commercially supplied pre-alloyed atomized powder, LaserForm 316L (A), from 3D Systems, Rock Hill, SC, USA, with a mean particle diameter of approximately 35 μm. All SLM fabrication experiments were carried out using a TB-PBFM-700 metal 3D printer, Wuhu, China. Because the thermophysical and mechanical properties of 316L stainless steel vary nonlinearly with temperature during selective laser melting, these temperature-dependent changes play a decisive role in determining the accuracy of the simulated thermal and stress fields. To account for this effect, temperature-specific material properties were assigned across different intervals within the model, as detailed in Table 2. This approach substantially improves the fidelity and robustness of the numerical predictions, providing a solid basis for the subsequent simulation analyses.

### 2.2. Finite Element Model

**Figure 1:**
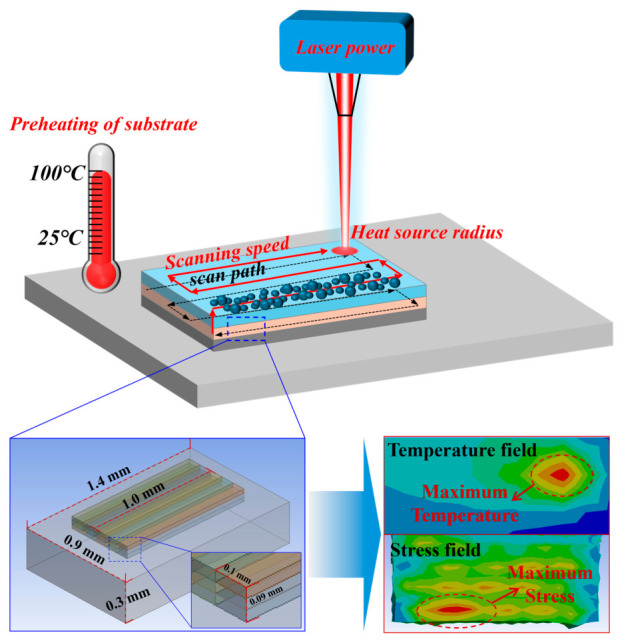
Finite element model of selective laser melting. The black dashed arrow indicates the scanning path of the first printing layer; the red solid arrow represents the upward printing path layer by layer; the red dashed circle denotes the concentration region of temperature and stress.

Numerical simulations of the temperature and stress fields were carried out in ANSYS Workbench 2022 using a transient thermal analysis, followed by stress-field evaluation based on the simulated thermal history [28,29]. The finite element model comprised five scan tracks and three deposited layers. The dimensions of the build region were 1 mm × 0.5 mm × 0.09 mm, and those of the substrate were 1.4 mm × 0.9 mm × 0.3 mm, as shown in Figure 1. A zigzag scanning strategy was adopted for the three-layer deposition along the z-direction, which is a standard scanning pattern widely employed in practical SLM manufacturing. To approximate the boundary constraints during fabrication, fixed supports were applied to the long sides of the substrate. A moving Gaussian heat-source subroutine was implemented in APDL, and the “element birth and death” technique was used to simulate the layer-by-layer deposition process of SLM [30,31]. The code provided in Appendix A is intended to illustrate the implementation logic of the moving Gaussian heat source for a representative case; the full simulation dataset was generated by varying the prescribed process parameters in a parameterized modeling workflow.

To balance computational accuracy with computational efficiency, several simplifying assumptions were introduced. First, the powder bed was treated as a homogeneous and continuous medium. Second, convection and radiation were represented in a simplified manner, and an equivalent enhanced convective heat-transfer coefficient was applied to the bottom surface to approximate the rapid heat dissipation provided by the massive build platform. Third, complex melt-pool fluid flow was neglected. Fourth, fluctuations in laser power and powder size were not considered. Fifth, an ultra-fine mesh of 10 μm was applied in the laser scanning region to capture the steep thermal gradients, whereas a coarser mesh was used for the substrate. Under these assumptions, the present model is intended primarily for comparative evaluation of process-parameter effects rather than for full high-fidelity reproduction of all physical mechanisms involved in SLM [32].

In the subsequent surrogate-modeling framework, four process parameters—laser power (LP), scanning speed (SS), heat-source diameter (HSD), and substrate preheating temperature (SPH)—were used as the input variables. The primary response quantity extracted from the simulation results was the simulated average thermal stress, *σ*_avg_. In the present study, *σ*_avg_ is derived from the von Mises equivalent stress, *σ*_e_, which is solved by ANSYS Mechanical based on the maximum distortion energy theory. The stress component form of *σ*_e_ is defined as [33]

(1) $$\sigma_{e}=\sqrt{\frac{1}{2}\left[{\left(\sigma_{x}-\sigma_{y}\right)}^{2}+{\left(\sigma_{y}-\sigma_{z}\right)}^{2}+{\left(\sigma_{z}-\sigma_{x}\right)}^{2}+6{\left({\tau_{\mathrm{xy}}}^{2}+{\tau_{\mathrm{yz}}}^{2}+{\tau_{\mathrm{zx}}}^{2}\right)}^{2}\right]}$$

where *σ*_x_, *σ*_y_, and *σ*_z_ are the normal stress components in the X, Y, and Z directions, respectively, and *τ*_xy_, *τ*_yz_, and *τ*_zx_ are the shear stress components acting on the XY, YZ, and ZX planes, respectively. To eliminate mesh sensitivity and local stress concentration effects, and to capture the overall thermo-mechanical response of the deposited region, *σ*_avg_ is further defined as the volume-weighted average of *σ*_e_ over all activated deposited elements at the final simulation step for a given process condition:

(2) $$\sigma_{\mathrm{avg}}=\frac{\sum_{i=1}^{N}\sigma_{e,i}V_{i}}{\sum_{i=1}^{N}V_{i}}$$

where *σ*_e,i_ is the von Mises equivalent stress of the i-th deposited element, V_i_ is the volume of the i-th element, and N is the total number of activated deposited elements.

It should be noted that von Mises stress is a scalar equivalent stress and does not preserve the sign, direction, or tensorial components of the stress state. Therefore, *σ*_avg_ does not represent the complete residual-stress tensor field, nor can it distinguish between tensile and compressive stress components or through-thickness stress gradients. In this work, *σ*_avg_ is used only as a simulation-derived comparative thermal stress indicator for ranking process conditions within the investigated parameter space. The subsequent comparison with XRD-derived macroscopic stress estimates is therefore conducted only at the trend level, not as a strict quantitative equivalence between the simulated *σ*_avg_ and experimentally measured residual stress.

### 2.3. Machine Learning

#### 2.3.1. Feature Selection

To construct a surrogate model for rapid comparative screening, a machine learning dataset was established based on the finite element simulation results. The input variables included four process parameters, namely laser power (LP), scanning speed (SS), heat-source diameter (HSD), and substrate preheating temperature (SPH). The primary output variable was the simulated average thermal stress, *σ*_avg_.

Before model training, Pearson correlation analysis was performed as a descriptive statistical tool to examine the pairwise linear relationships among the input variables and the target variable. Let the data matrix be X = (x*_ij_*)*_m_*_×*n*_, where each column corresponds to one feature. The Pearson correlation coefficient between the *a*-th and *b*-th column features is defined as follows:

(3) $$\rho (a,b)=\frac{\sum_{i=1}^{m}{(x}_{a,i}-\bar{x_{a}}{)(x}_{b,i}-\bar{x_{b}})}{\sqrt{\sum_{i=1}^{m}{{(x}_{a,i}-\bar{x_{a}})}^{2}}\sqrt{\sum_{i=1}^{m}{{(x}_{b,i}-\bar{x_{b}})}^{2}}}$$

where $\bar{x}$_a_ and $\bar{x}$_b_ denote the means of the *a*-th and *b*-th column features, respectively. The correlation coefficient ranges from [−1, 1], where −1 indicates a perfect negative linear correlation, 0 indicates no linear correlation, and 1 indicates a perfect positive linear correlation.

It should be noted that the Pearson correlation reflects only pairwise linear dependence. Because the present response is governed by nonlinear and coupled thermo-mechanical interactions, Pearson analysis was used here only for preliminary data description rather than as a strict feature-selection criterion. Therefore, all four process parameters were retained for subsequent model training and interpretation.

#### 2.3.2. Data Preprocessing

The raw finite-element-generated dataset initially contained 598 samples. During the preliminary data-quality inspection, 30 samples were excluded before model development because they showed abnormal numerical-output behavior relative to the physically reasonable response range of the retained dataset. These excluded cases corresponded to simulations that were not considered sufficiently reliable for surrogate-model training. The remaining 568 samples were used for the subsequent machine learning analysis and were randomly divided into training, validation, and test subsets with a ratio of 8:1:1, corresponding to 454, 57, and 57 samples, respectively.

To further examine the robustness of the retained dataset, Isolation Forest was used as an auxiliary diagnostic tool after data partitioning. The anomaly detection model was fitted using the training subset only and was used to examine whether the retained samples still contained additional potentially abnormal cases. In the present study, this procedure was used as a diagnostic check rather than as a second-stage automatic deletion rule. Unless otherwise stated, all retained samples were used in the subsequent regression analysis.

After data inspection, all input features and target values were standardized using Z-score normalization. The normalization parameters, including the mean and standard deviation, were estimated from the training subset only and then applied to the validation and test subsets. This procedure avoids information leakage and ensures that the reported model performance reflects prediction quality within the investigated simulation-generated dataset.

#### 2.3.3. Regression Models

All machine learning algorithms were implemented in MATLAB [34,35]. Nine supervised regression models were considered in this study: Multiple Linear Regression (MLR), Generalized Additive Model (GAM), Gaussian Kernel Regression (GKR), Gaussian Process Regression (GPR), Decision Tree Regression (DTR), Random Forest Regression (RF), LSBoost, Backpropagation Neural Network (BPNN), and Long Short-Term Memory (LSTM) [36,37]. These models were selected to represent different categories of regression approaches, including conventional statistical models, kernel-based models, tree-based ensemble models, and neural network-based models. Their predictive performance was compared systematically to identify the most suitable surrogate model for the present simulation dataset of *σ*_avg_.

#### 2.3.4. Hyperparameter Optimization and Model Evaluation

Hyperparameters were optimized using Bayesian optimization with up to 30 objective evaluations. Model performance was evaluated using mean absolute error (MAE), mean absolute percentage error (MAPE), mean squared error (MSE), root mean squared error (RMSE), and the coefficient of determination ($R^{2}$) [38]. These metrics are defined as follows:

(4) $$\mathrm{MAE}=\frac{1}{n}\sum_{i=1}^{n}\left|y_{i}-{\hat{y}}_{i}\right|$$

(5) $$\mathrm{MAPE}=\frac{1}{n}\sum_{i=1}^{n}\left|\frac{y_{i}-{\hat{y}}_{i}}{y_{i}}\right|$$

(6) $$\mathrm{MSE}=\frac{1}{n}\sum_{i=1}^{n}{\left(y_{i}-{\hat{y}}_{i}\right)}^{2}$$

(7) $$\mathrm{RMSE}=\sqrt{\frac{1}{n}\sum_{i=1}^{n}{\left(y_{i}-{\hat{y}}_{i}\right)}^{2}}$$

(8) $$R^{2}=1\;-\;\frac{\sum_{i=1}^{n}{\left(y_{i}-{\hat{y}}_{i}\right)}^{2}}{\sum_{i=1}^{n}{\left(y_{i}-\bar{y_{i}}\right)}^{2}}$$

where n is the number of data points and $y_{i}$, ${\hat{y}}_{i}$, and $\bar{y}$ represent the actual value, predicted value, and mean of the actual values, respectively.

For reproducibility, the representative BPNN model was implemented in MATLAB R2021b using the fitrnet function. A two-hidden-layer structure with 16 neurons in each hidden layer, i.e., ref. [16], was adopted as the representative network architecture in the present study, and the regularization-related hyperparameters were optimized using Bayesian optimization with the validation error as the objective function. All models were trained and evaluated under the same training/validation/test split to ensure a fair comparison within the present dataset.

It should be emphasized that the validation subset was used for hyperparameter tuning and model selection, while the test subset was reserved for one-time performance reporting. Therefore, the reported results should be interpreted as evidence of strong predictive accuracy within the investigated simulation space rather than as proof of universal out-of-sample generalization to arbitrary SLM conditions.

#### 2.3.5. Model Selection Strategy

The nine regression models were first compared in terms of predictive accuracy on the training, validation, and test subsets. BPNN exhibited the best overall predictive performance among the evaluated models. However, because the present problem is a structured, static tabular regression task rather than a sequential time-series task, BPNN was selected as the final representative surrogate model for subsequent interpretation and mechanism-oriented analysis. In the present context, BPNN offers three practical advantages: (1) predictive accuracy comparable to the best-performing models; (2) stable behavior across the retained dataset; and (3) direct applicability to feature-level interpretation using PDP and SHAP. Therefore, LSTM was retained as a high-performance comparison baseline, whereas BPNN was adopted as the main model for interpretation, stability assessment, and convergence analysis. This choice should be understood as a model-selection decision for the present dataset rather than as general evidence that recurrent neural network architectures are preferable for static additive manufacturing parameter datasets.

### 2.4. Experimental Support for the Simulation-Guided Screening Results

To provide limited experimental support for the dominant trends identified by the simulation-guided screening framework, 316L stainless steel powder was used as the feedstock, and cubic specimens measuring 1 cm × 1 cm × 0.8 cm were fabricated using an SLM system [39]. Because the machine learning interpretation identified laser power and scanning speed as the dominant variables within the present dataset, the experimental campaign focused on these two process parameters. A total of 14 forming experiments were conducted, as summarized in Table 3. The laser power ranged from 120 to 240 W with an interval of 20 W, while the scanning speed was set at two levels, 950 mm/s and 1050 mm/s. The remaining process settings, including heat-source diameter and substrate preheating temperature, were kept constant at 100 μm and 100 °C, respectively. Accordingly, the experiments were designed to provide targeted support for the dominant LP–SS trends identified by the model, rather than a complete four-factor validation of the surrogate framework.

Metallographic microscopy (DMM-900C, Shanghai Caikon Optical Instrument Co., Ltd., Shanghai, China) was used to characterize the forming quality and microstructural morphology of the fabricated specimens at magnifications of 50×, 100×, 400×, and 600×. Prior to observation, the samples were mechanically polished and chemically etched to ensure sufficient contrast. In the present work, the metallographic observations are used primarily as qualitative evidence of relative forming quality under different process conditions rather than as a quantitative validation of the simulated stress descriptor.

XRD analysis of the *γ*(220) peak position was further performed to derive an approximate macroscopic residual-stress estimate. For each process condition, three repeated XRD measurements were conducted, and the mean value and standard deviation were calculated to obtain the average macroscopic stress estimate and the corresponding error bar. According to the relationship between diffraction peak shift and lattice strain, the lattice strain ε was determined using Equation (9), and a corresponding stress estimate was obtained under a simplified elastic assumption using Equation (10). The elastic modulus and Poisson’s ratio of 316L stainless steel were taken as E = 200 GPa and ν = 0.30, respectively [40,41]. It should be emphasized that the present XRD analysis is based on the γ(220) peak shift relative to the standard Bragg angle and is used as an approximate macroscopic stress indicator. Accordingly, the XRD-derived stresses are compared with the simulated *σ*_avg_ values only at the level of variation trend rather than strict quantitative equivalence.

(9) $$\varepsilon =\frac{\Delta d}{d_{0}}=-\cot\theta_{0}\cdot \Delta \theta$$

where d_0_ is the standard interplanar spacing of the γ(220) plane, *θ*_0_ is the standard Bragg angle (74.697°), Δ*θ* is the peak shift (Δ2*θ*/2), and Δd represents the change in interplanar spacing.

Based on the generalized Hooke’s law, the macroscopic residual stress *σ* can be further calculated from the lattice strain:

(10) $$\sigma =\;\frac{E}{1+\nu }\cdot \varepsilon$$

where E is the elastic modulus of 316L stainless steel, and ν is the Poisson’s ratio. In this study, the residual stress value was directly obtained from the measured peak shift Δ2*θ* between the experimental 2*θ* and the standard 2*θ* (74.697°) of the *γ*(220) peak.

## 3. Results

### 3.1. Influence of Process Parameters on Simulated Thermal Stress Indicators and Deformation

#### 3.1.1. Analysis of Temperature Field and Stress Field

**Figure 2:**
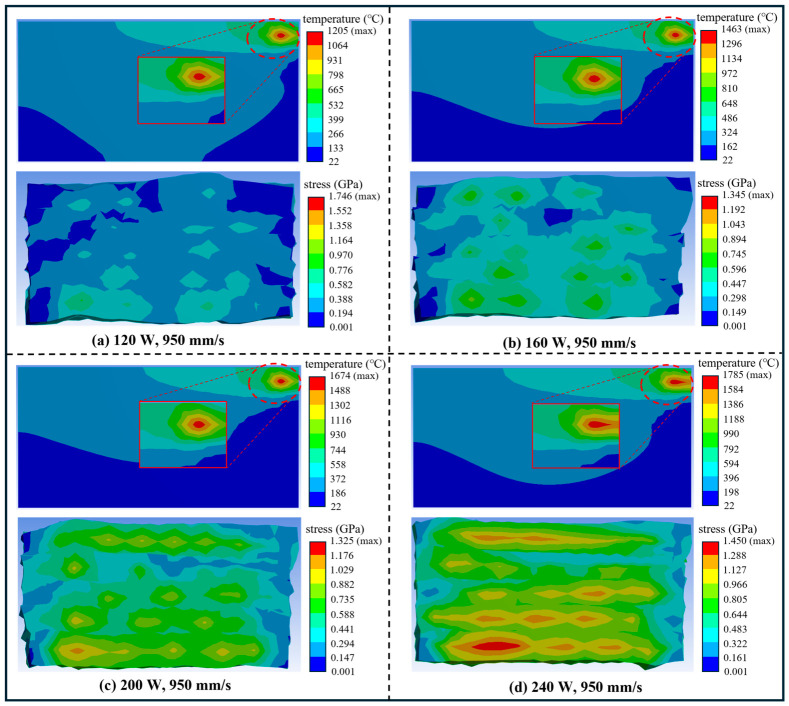
Representative simulated temperature fields and stress fields under different process conditions.

As shown in Figure 2a–d, the selected monitoring point corresponds to a stage close to the end of the simulation. The simulations were conducted at a fixed scanning speed of 950 mm/s, an HSD of 100 μm, and a substrate temperature of 22 °C, with laser powers of 120, 160, 200, and 240 W. The simulated peak temperature increased with increasing laser power, from 1205 °C at 120 W to 1785 °C at 240 W, indicating enhanced thermal input at higher powers. Accordingly, lower laser power results in a lower peak temperature, whereas higher laser power leads to a further temperature increase. The internal stress exhibited a non-monotonic response to laser power. The highest stress occurred at 120 W due to insufficient energy input, decreased to a minimum at 200 W, and then increased again at 240 W, likely because of enhanced thermal gradients and heat accumulation. These temperature and stress trends should be regarded as model-specific responses under the present simulation conditions rather than universal rules for all SLM systems [42,43].

**Figure 3:**
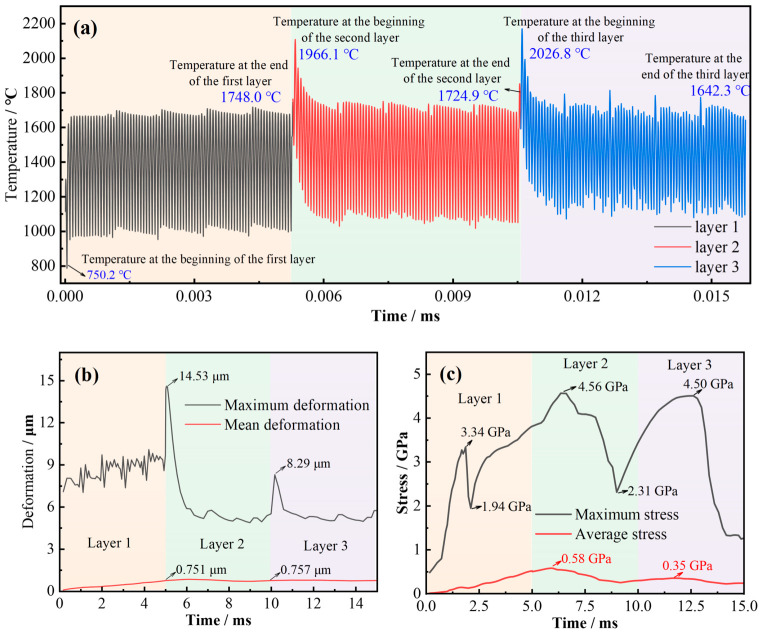
Simulated evolution of (**a**) temperature, (**b**) deformation, and (**c**) stress during a representative SLM process.

Representative process parameters were adopted in this study, including an LP of 200 W, SS of 950 mm/s, HSD of 100 μm, and a substrate temperature of 22 °C. The corresponding numerical results are presented in Figure 3, which demonstrates that the temperature, deformation, and stress exhibit pronounced layer-dependent evolution throughout the SLM process [44,45]. Figure 3a shows that the temperature fluctuates periodically with layer deposition, reflecting the transient thermal cycles induced by alternating laser heat input and rapid cooling, while the deposition of subsequent layers also produces a clear reheating effect on the previously formed region. Specifically, the temperature at the beginning of the first, second, and third layers reached 750.2 °C, 1966.1 °C, and 2026.8 °C, respectively. The temperature at the end of the first, second, and third layers reached 1748.0 °C, 1724.9 °C, and 1642.3 °C, respectively. Figure 3b indicates that deformation accumulates progressively during deposition and increases markedly at interlayer transitions, suggesting that the deformation response is jointly governed by thermal strain accumulation and structural constraint. The maximum deformation reached 14.53 μm in Layer 2 and 8.29 μm in Layer 3, whereas the mean deformation remained much lower, at 0.751 μm and 0.757 μm, respectively. Figure 3c shows that the stress evolution is strongly nonlinear: the instantaneous maximum stress characterizes local transient stress concentration, whereas the average stress remains lower and more stable, making it more suitable for describing the overall stress evolution and for subsequent trend analysis. In particular, the simulated maximum stress reached 3.34 GPa and 1.94 GPa in Layer 1, 4.56 GPa and 2.31 GPa in Layer 2, and 4.50 GPa in Layer 3, while the average stress remained within approximately 0.3–0.6 GPa, with representative values of 0.58 GPa and 0.35 GPa.

**Figure 4:**
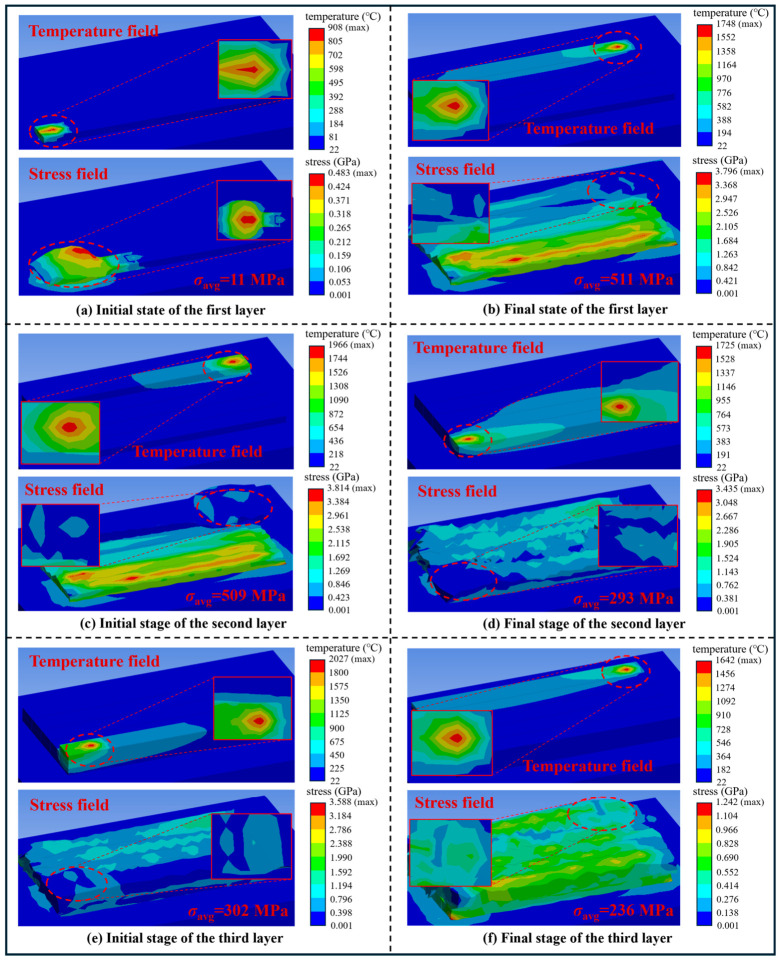
Evolution of temperature and stress fields during three-layer SLM deposition at 200 W laser power and 950 mm/s scanning speed.

Figure 4 presents the typical temperature and stress field distributions at the onset and completion of the first three deposition layers under the SLM conditions of 200 W laser power and 950 mm/s scanning speed, corresponding to the representative case illustrated in Figure 3. To facilitate high-throughput multi-parameter numerical simulations, the thermo-mechanical model adopted in this work possesses lower physical comprehensiveness relative to state-of-the-art high-fidelity residual stress simulation models. Despite this limitation, the proposed model can effectively characterize the key qualitative evolution laws of thermo-mechanical behavior during the SLM manufacturing process. The temperature in the laser-irradiated zone exceeds the melting point of 316L stainless steel, leaving the molten powder in a low-stress state, while the stress in the scanned region increases significantly upon cooling and solidification, which is fully consistent with the physical behavior of actual SLM processes. The interlayer stress evolution follows a distinct pattern: as shown in Figure 4a,b, the initial average stress of the first layer is merely 11 MPa and accumulates to 511 MPa after cooling at the end of deposition; as presented in Figure 4c,d, affected by the residual stress inherited from the first layer, the initial average stress of the second layer reaches 509 MPa and relaxes to 293 MPa at the end of the layer; as displayed in Figure 4e,f, the third layer maintains a continuous stress relaxation trend with an initial stress of 302 MPa and a final average stress of 236 MPa upon completion. The stress distribution reveals that the first layer possesses a relatively high overall stress level; the energy input during the second-layer deposition reduces the thermal gradient of the system, effectively relieving the high stress in the first layer and lowering the global stress level accordingly. The instantaneous average stress varies noticeably at different moments. The global average stress *σ*_avg_, calculated using the volume-weighted formulation after the full simulation, effectively circumvents the mesh dependence of local peak stresses and objectively characterizes the global average stress state of the as-built component. This indicator is highly consistent with the macroscopic residual stress measured by XRD in physical connotation: both reflect the bulk average stress of the component rather than localized peak stresses, enabling a reliable trend-level correlation with conventional residual stress measurements. The results validate that *σ*_avg_ can reliably characterize the macroscopic stress level of SLM-fabricated parts and meet the requirements for process parameter screening and experimental correlation.

**Figure 5:**
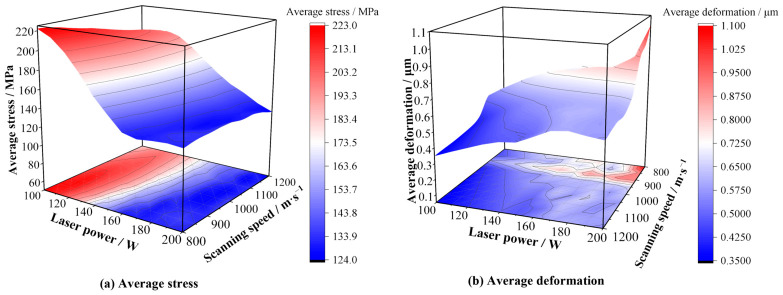
Representative response surfaces showing the influence of selected input parameters on simulated *σ*_avg_ and maximum deformation.

The response surfaces in Figure 5 further show that laser power exerts the strongest influence on the simulated stress indicator and deformation. Over the investigated range, the *σ*_avg_-based response is non-monotonic, with relatively high values in the low-power region and substantially lower values near the intermediate- to high-power region around 180–200 W. By contrast, deformation increases more clearly under higher laser power and lower scanning speed. Taken together, these results suggest that the favorable process window in the present study should be understood as a compromise among average thermal stress level, deformation, and overall forming quality under the assumptions of the adopted model.

#### 3.1.2. Effects of Laser Power on the *σ*_avg_-Based Thermal Stress Response

**Figure 6:**
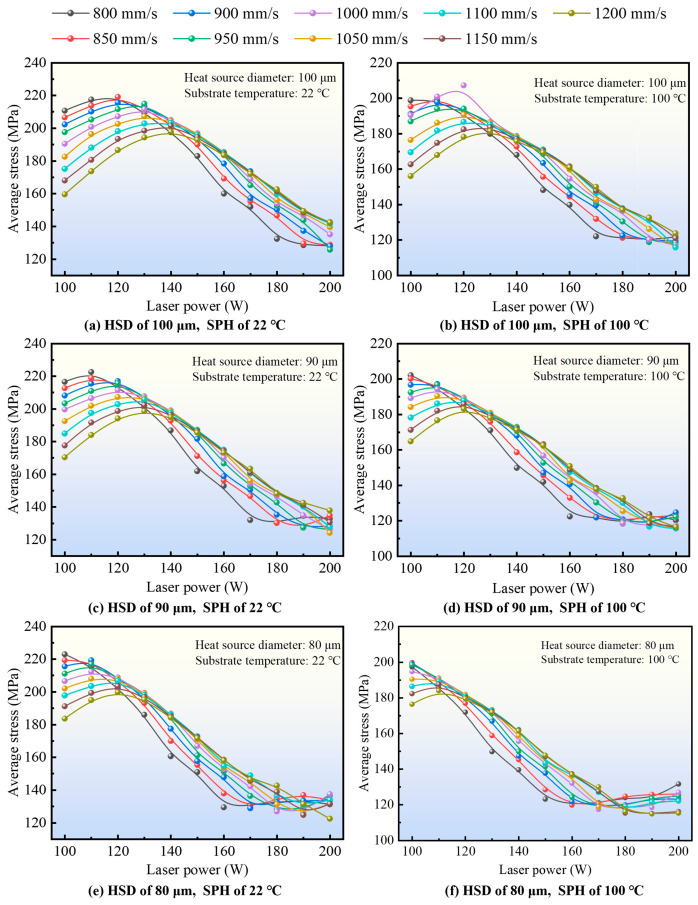
The effect of laser power on the *σ*_avg_-based thermal stress response.

Figure 6 illustrates the effect of laser power on the simulated *σ*_avg_-based thermal stress response during SLM processing. Overall, laser power exerts the strongest influence among the investigated variables. In the low-power range (100–140 W), the predicted *σ*_avg_ remains at a relatively high level, indicating a less favorable thermal–mechanical state under insufficient energy input. As the laser power increases into the intermediate-to-high range, the predicted *σ*_avg_ decreases substantially and reaches a lower level near approximately 180–200 W for most of the investigated parameter combinations. Within the present process window, this trend suggests that the thermal input becomes more favorable for reducing the overall average stress level at these power levels.

A comparison of different substrate temperatures further shows that preheating the substrate from 22 °C to 100 °C reduces the overall *σ*_avg_ response within the same laser-power interval. In addition, decreasing the heat-source diameter from 100 μm to 80 μm also modifies the response, although its influence remains secondary relative to that of laser power. Taken together, these results indicate that laser power is the dominant control variable in the present dataset, while substrate preheating and heat-source diameter play secondary but non-negligible roles in modifying the average thermal stress response [46,47]. Substrate preheating to 100 °C raises the overall initial temperature of the workpiece, narrows the temperature difference between the laser scanning zone and other workpiece regions, and thus effectively lowers the average thermal stress *σ*_avg_.

It should be emphasized that the present discussion is based on the comparative behavior of the simulated *σ*_avg_ index. Therefore, the above trends should be interpreted as relative screening results within the investigated parameter range.

#### 3.1.3. Effects of Scanning Speed on the *σ*_avg_-Based Thermal Stress Response

**Figure 7:**
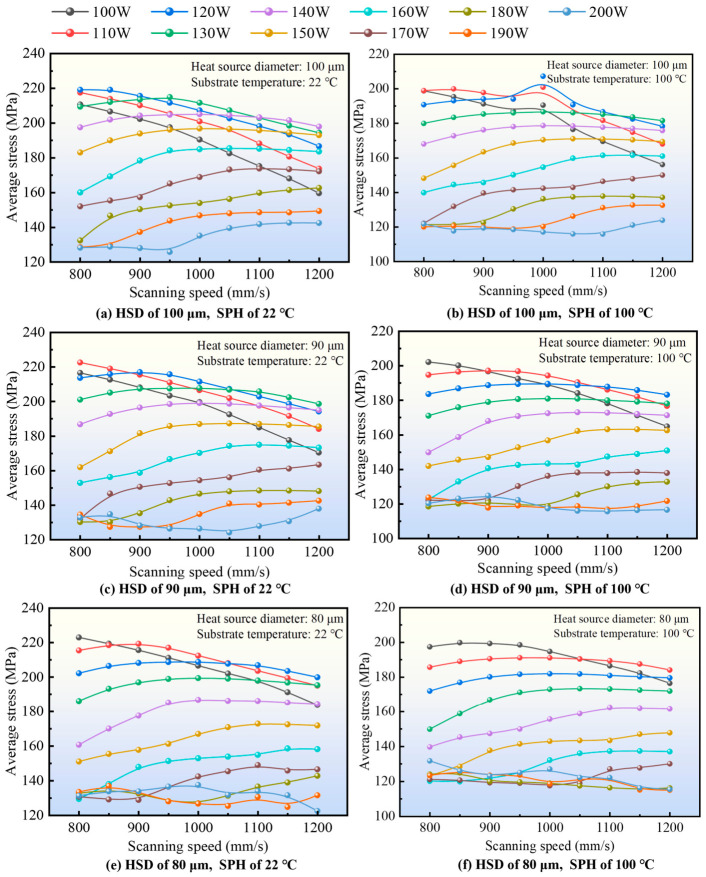
The effect of scanning speed on the *σ*_avg_-based thermal stress response.

Figure 7 shows the influence of scanning speed on the simulated *σ*_avg_-based thermal stress response. Compared with laser power, the effect of scanning speed is weaker and more dependent on coupled parameter conditions. At a substrate temperature of 22 °C, increasing the laser power from 100 W to 200 W generally reduces the predicted *σ*_avg_, whereas the effect of scanning speed varies with the laser power level and does not follow a single, strictly monotonic pattern across the full parameter window. This indicates that scanning speed mainly acts as a secondary modulation factor relative to laser power in the present dataset.

When the substrate temperature is increased to 100 °C, the overall level of the predicted *σ*_avg_ is further reduced for most parameter combinations. In addition, the heat-source diameter also affects the shape of the response surface, although its contribution remains weaker than that of laser power. These observations suggest that scanning speed influences the average thermal stress response mainly through its coupling with energy input and heat accumulation, whereas laser power remains the dominant governing variable [48,49].

Overall, the results of Figure 6 and Figure 7 consistently indicate that the lower average thermal stress level in the present study is obtained near the 200 W region, although the exact response still depends on the coupled effects of scanning speed, heat-source diameter, and substrate preheating temperature. Therefore, the identified favorable condition should be understood as a local optimum within the investigated process window.

### 3.2. Machine Learning Results

#### 3.2.1. Data Processing

**Figure 8:**
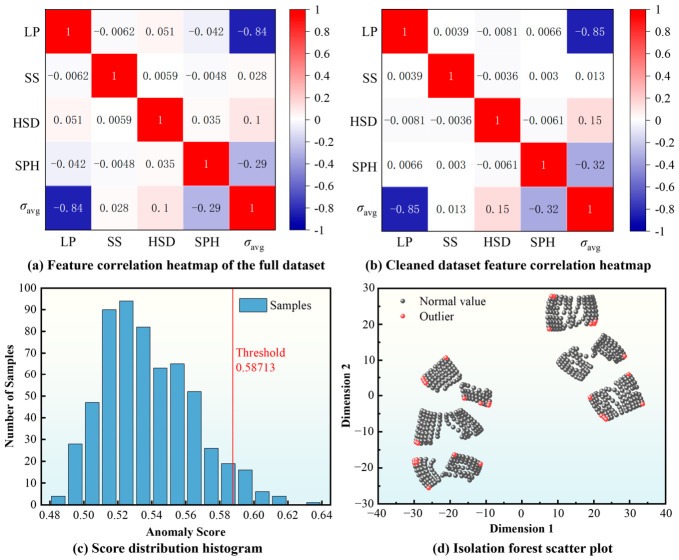
Feature-correlation analysis and diagnostic outlier inspection of the retained dataset.

To characterize the relationships between process parameters and the simulated average thermal stress, preliminary correlation analysis and data-quality inspection were performed, as shown in Figure 8. Figure 8a,b present feature-correlation heatmaps for the full dataset and the cleaned dataset, respectively. After outlier removal, the Pearson correlation coefficient between LP and *σ*_avg_ strengthened from −0.84 to −0.85, and the correlation between SPH and *σ*_avg_ became more pronounced, changing from −0.29 to −0.32, indicating improved physical consistency of the cleaned dataset. The feature-correlation heatmap in Figure 8b indicates that LP exhibits the strongest negative linear correlation with *σ*_avg_, suggesting that increasing LP tends to reduce the *σ*_avg_-based response within the investigated parameter range. By contrast, SS, HSD, and SPH show weaker pairwise linear correlations with the target variable.

However, because the thermal stress response in SLM is governed by nonlinear and coupled thermo-mechanical effects, Pearson correlation was used here only as a descriptive statistical tool and not as a strict feature-selection criterion. Accordingly, all four process parameters were retained for subsequent model training and interpretation.

Figure 8c,d show the diagnostic results based on the Isolation Forest algorithm for the retained dataset. In Figure 8c, the histogram represents the distribution of anomaly scores, while the red vertical line denotes the threshold of 0.58713 (the 95th percentile of the score distribution) used to flag potentially abnormal retained cases for further inspection. Figure 8d provides a three-dimensional visualization of the retained samples and the flagged cases. It should be emphasized that this procedure was used only as an auxiliary diagnostic step after the preliminary exclusion of 30 abnormal samples described in Section 2.3.2, rather than as the primary basis for sample deletion [50].

In summary, the data-processing procedure, including preliminary correlation analysis, pre-screening of abnormal numerical outputs, diagnostic anomaly inspection, and standardized normalization, established a consistent basis for developing the machine learning surrogate models for *σ*_avg_ prediction.

#### 3.2.2. Comparison of Machine Learning Algorithms

**Figure 9:**
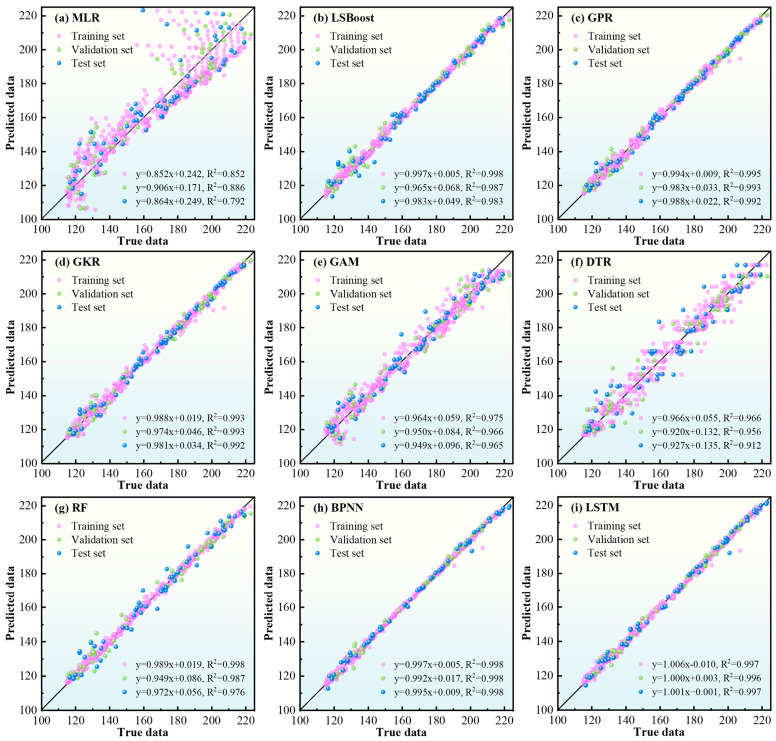
Comparison of regression performance for nine machine learning models.

The predictive performance of the nine regression models was evaluated on the training, validation, and test subsets. As shown in Figure 9, BPNN and LSTM achieved the best overall fitting performance among the evaluated models. Both models yielded very high R^2^ values on the training and validation subsets. Nevertheless, BPNN achieves higher prediction accuracy and stability. The R^2^ values of BPNN for the training, validation and test sets are 0.998, 0.998 and 0.998, slightly higher than those of LSTM (0.997, 0.996 and 0.997). These results indicate that both neural network-based models can accurately approximate the nonlinear mapping between the process parameters and the finite-element-generated *σ*_avg_ response within the present simulation space.

By contrast, linear and weakly nonlinear models such as MLR, GAM, and DTR showed lower predictive accuracy, indicating a limited ability to capture the coupled nonlinear characteristics embedded in the dataset. Kernel-based and ensemble models, including GPR, GKR, LSBoost, and RF, performed better than the linear models, but still remained slightly inferior to BPNN in terms of overall predictive accuracy on the retained dataset.

The detailed error metrics are summarized in Table 4. For BPNN, the MAE, MSE, and RMSE values remained low across the training, validation, and test subsets, indicating strong fitting and general interpolation capability within the investigated dataset. LSTM showed similarly strong performance. In quantitative comparison, BPNN also has lower comprehensive error levels than LSTM. The MAE, MAPE and RMSE of BPNN are 1.038, 0.007 and 1.500, while those of LSTM are 1.101, 0.007 and 1.575, respectively. Nevertheless, because the target values were generated from a specific finite element model rather than from large-scale noisy experimental observations, these metrics should be interpreted as evidence of strong interpolation capability within the current simulation space rather than as unconditional proof of universal generalization to arbitrary SLM conditions.

The five-fold cross-validation MAE results also indicate that BPNN exhibits relatively stable error behavior within the present dataset. Considering the structured static tabular nature of the problem and the subsequent need for feature-level interpretation, BPNN was selected as the representative surrogate model for further analysis.

#### 3.2.3. BPNN-Based Model Interpretation and Stability Analysis

The choice of BPNN was motivated by three considerations: (1) predictive performance comparable to the best-performing model class in the present dataset; (2) stable error behavior across the retained dataset and cross-validation evaluation; and (3) direct applicability to feature-level interpretation in the present structured, static tabular problem.

**Figure 10:**
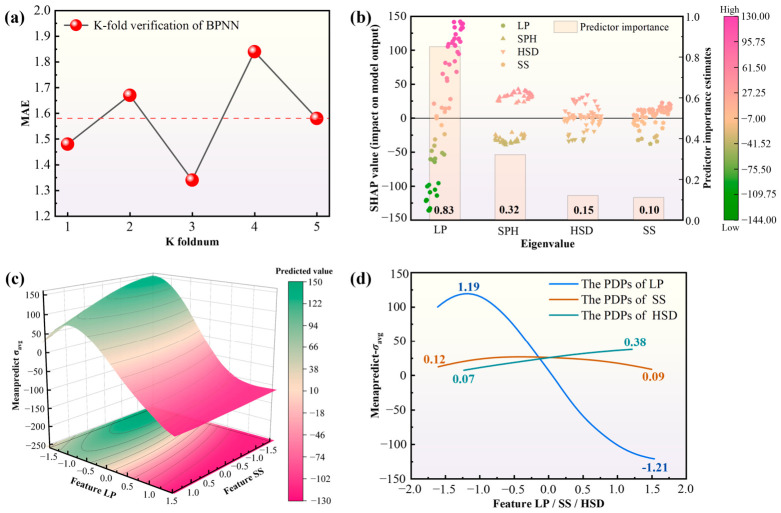
PDP and SHAP analyses of key features based on the representative BPNN model. (**a**) K-fold cross-validation (MAE) of the BPNN model; (**b**) SHAP summary plot and predictor importance of LP, SPH, HSD, and SS; (**c**) 3D PDP showing the joint effect of LP and SS on predicted average stress; (**d**) 1D PDPs showing the marginal effects of LP, SS, and HSD on predicted average stress.

As shown in Figure 10, the BPNN model exhibited stable predictive behavior within the investigated simulation space. To further interpret the learned nonlinear mapping, PDP and SHAP analyses were performed. The SHAP results indicate that laser power is the most influential variable, followed by substrate preheating temperature, whereas scanning speed and heat-source diameter contribute mainly through secondary or coupled effects. The PDP results further reveal nonlinear dependence and interaction effects, especially between laser power and scanning speed, indicating that the *σ*_avg_ response cannot be adequately described by a simple linear or single-parameter relationship.

It should be emphasized that the above interpretation reflects the response structure learned by the surrogate model within the present finite-element-generated dataset and parameter window. Therefore, the SHAP and PDP results should be interpreted as model-based insights into the investigated simulation space rather than as universal physical laws applicable to all SLM systems.

### 3.3. Experimental Support for the Simulation-Guided Parameter Screening

**Figure 11:**
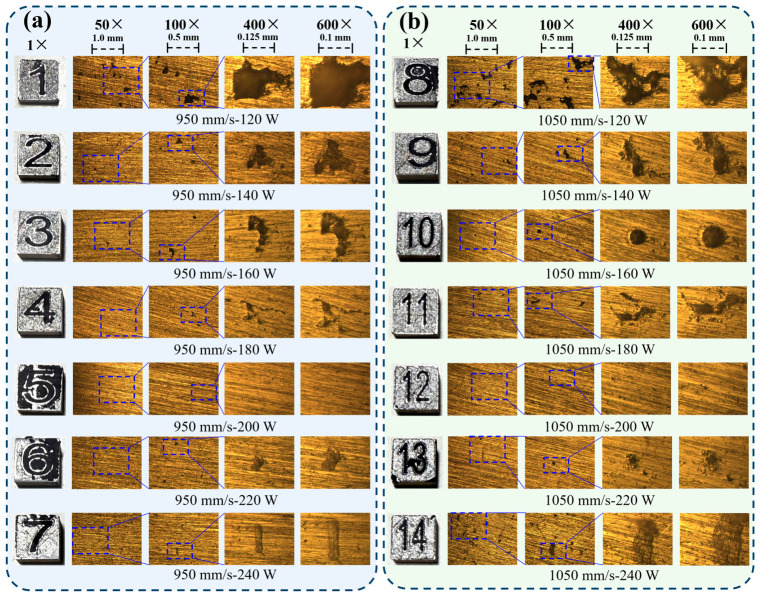
Metallographic observations of samples fabricated under different laser powers and scanning-speed conditions. (**a**) Metallographic images at a scanning speed of 950 mm/s under different laser powers; (**b**) Metallographic images at a scanning speed of 1050 mm/s under different laser powers.

As shown in Figure 11, the specimens fabricated at lower laser powers (120–160 W) under both scanning speeds exhibited pronounced porosity and irregular unmelted regions, indicating insufficient melting and inferior forming quality. By contrast, the specimens fabricated near 200 W showed a denser and more continuous morphology with visibly fewer defects. These metallographic observations are qualitatively consistent with the simulation-guided screening result that the *σ*_avg_-based thermal stress level is reduced in this power range. Nevertheless, the metallographic evidence should be regarded only as qualitative support for the parameter-screening outcome rather than as a one-to-one quantitative validation of the simulated *σ*_avg_ value.

**Figure 12:**
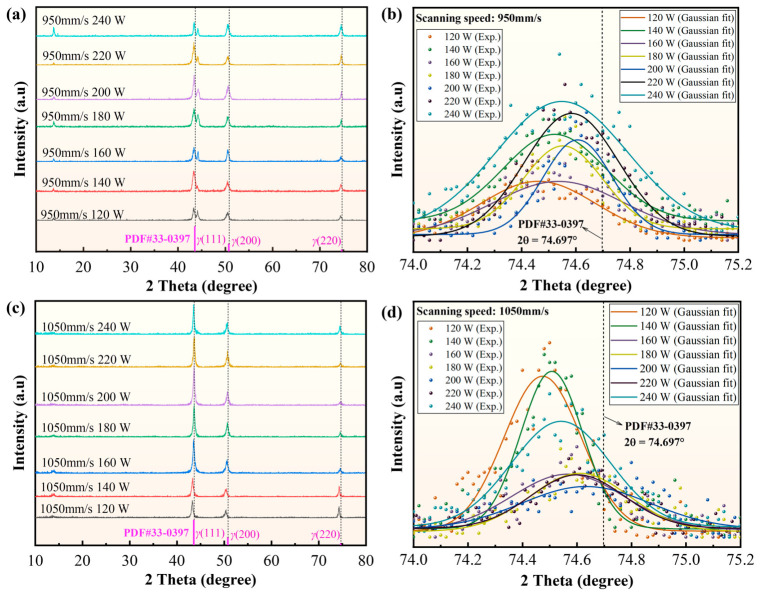
Representative XRD patterns and Gaussian fitting of the γ(220) crystal plane for SLM-fabricated 316L stainless steel under different process parameters. (**a**) XRD patterns at 950 mm/s under different powers; (**b**) Gaussian fitting of the 74–75.2° peak at 950 mm/s; (**c**) XRD patterns at 1050 mm/s under different powers; (**d**) Gaussian fitting of the 74–75.2° peak at 1050 mm/s.

To further examine the macroscopic stress state, XRD measurements were performed and the *γ*(220) peak shift was used to derive an approximate macroscopic residual-stress estimate. For each process condition, three repeated XRD measurements were conducted. The mean peak position was used to calculate the average stress estimate, and the standard deviation was used to construct the corresponding error bar. As shown in the representative XRD results in Figure 12, all specimens maintained an austenitic γ-phase structure, and the diffraction peaks shifted toward lower angles, indicating tensile residual-stress tendencies. The derived stress values ranged from approximately 70 to 210 MPa depending on the process parameters. Because these XRD-based results correspond to simplified post-process macroscopic stress estimates obtained from repeated measurements under each condition, they are used here only for trend-level comparison with the simulated *σ*_avg_ values. Detailed XRD results are provided in Appendix B.

**Figure 13:**
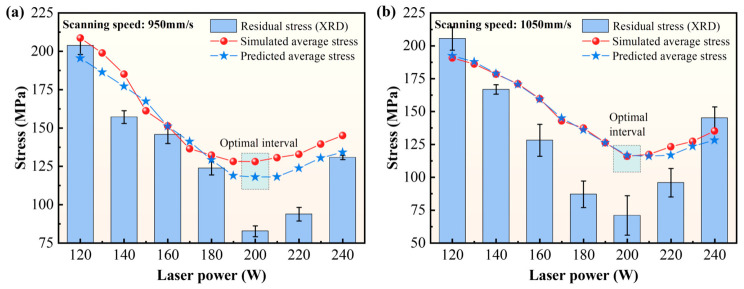
Trend-level comparison between XRD-derived macroscopic stress estimates and simulated *σ*_avg_ at scanning speeds of 950 mm/s and 1050 mm/s. (**a**) Comparison at a scanning speed of 950 mm/s; (**b**) Comparison at a scanning speed of 1050 mm/s.

The calculated XRD-based stress estimates are summarized in Table 5. As illustrated in Figure 13, the XRD-derived macroscopic stress estimates and the simulated *σ*_avg_ values exhibited similar variation trends at both scanning speeds of 950 mm/s and 1050 mm/s. Error analysis was further conducted based on the standard deviation (SD) of residual stress from repeated XRD tests. The SD values range from 1.34 to 14.99 MPa, as summarized in Table 5. These relatively small deviations indicate good repeatability of the XRD-based stress estimates and further improve the credibility of the experimental results. In both cases, the stress level decreased with increasing laser power and reached its minimum within the 180–200 W range, which can be regarded as the local optimum in the investigated parameter window. The minimum XRD-derived stress values were 82.68 MPa at 950 mm/s and 70.96 MPa at 1050 mm/s. As laser power increased beyond 200 W, the stress level rose again, reaching 130.773 MPa at 950 mm/s and 145.14 MPa at 1050 mm/s under 240 W. Moreover, for the same laser power, the 1050 mm/s condition generally showed a higher stress level than the 950 mm/s condition. The influence of scanning speed on residual stress is far less significant than that of laser power, and the minimum stress occurs at 180–200 W, which is consistent with the defect evolution observed from metallographic results. Overall, the XRD-derived stress estimates ranged from 82.68 to 205.54 MPa, with corresponding stress standard deviations reported in Table 5. Taken together, the metallographic observations and XRD results provide targeted experimental support for the practical usefulness of the proposed framework in identifying favorable parameter windows, especially with respect to the dominant variables LP and SS.

However, because the experiments were conducted only for LP and SS while HSD and SPH were kept constant, the 180–200 W region is identified as a local optimum within the studied parameter window. The present results should be interpreted as support for the dominant two-factor trend within the tested LP–SS window rather than as a full experimental validation of the four-factor *σ*_avg_-based surrogate model, due to the lack of experimental validation for HSD and SPH.

**Figure 14:**
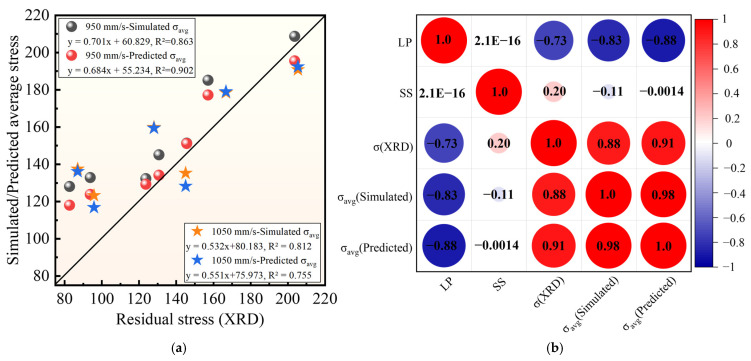
Validation and correlation analysis of simulated/predicted residual stress against XRD measurements. (**a**) Simulated/measured residual stress comparison; (**b**) Correlation analysis of residual stress results, where the size of each circle represents the magnitude of the correlation coefficient between two variables.

Figure 14 further compares the XRD-derived macroscopic residual-stress estimates with the simulated average thermal stress, *σ*_avg_, at 950 mm/s and 1050 mm/s. As shown in Figure 14a, both the simulated *σ*_avg_ and the BPNN-predicted *σ*_avg_ exhibit positive trend-level correlations with the XRD-derived macroscopic stress estimates under the tested LP–SS conditions. The corresponding R^2^ values are 0.863 and 0.902 at 950 mm/s, and 0.812 and 0.755 at 1050 mm/s, respectively. These results suggest that both the finite element simulation and the machine learning surrogate model can capture the dominant LP-dependent stress variation trend within the investigated LP–SS parameter range. However, this agreement should be understood as consistency in relative process ranking rather than as direct quantitative validation of the complete residual-stress field.

The Pearson correlation heatmap in Figure 14b further verifies such trend-level consistency. Laser power has strong negative correlations with the XRD-derived macroscopic stress estimate, simulated *σ*_avg_, and BPNN-predicted *σ*_avg_, with correlation coefficients of −0.73, −0.83, and −0.88, respectively. This result confirms that laser power acts as the dominant factor governing the stress response within the investigated parameter window. Moreover, the correlation coefficients between the XRD-derived macroscopic stress estimate and the simulated/BPNN-predicted *σ*_avg_ reach 0.88 and 0.91. These favorable correlations further validate that *σ*_avg_ can be adopted as a simulation-based comparative descriptor for screening stress variation trends.

Nevertheless, these correlations should be interpreted as trend-level agreement rather than strict quantitative equivalence, owing to the simplified simulation assumptions and the uncertainty associated with XRD-based residual-stress estimation.

## 4. Discussion

This study proposes a finite element simulation-driven machine learning framework for rapid comparative screening in SLM-fabricated 316L stainless steel. The main contribution of the work lies in combining simulation-generated process-response data with an interpretable surrogate model, thereby reducing the computational burden associated with repeated transient thermo-mechanical simulations over a multi-parameter design space. The finite element model used here should be regarded as a simplified thermo-mechanical model for efficient data generation, rather than a high-fidelity residual-stress model [51]. Compared with state-of-the-art simulations that may include full thermo-elasto-plastic constitutive behavior, stress relaxation, powder-scale heat transfer, melt-pool flow, and more detailed convection/radiation treatments, the present model adopts a homogeneous powder-bed approximation, simplified thermal boundary conditions, and neglects melt-pool hydrodynamics [52,53]. Therefore, its physical completeness is lower than that of many advanced residual-stress simulations, and its purpose is comparative process screening rather than quantitative reconstruction of the full residual-stress field. Importantly, *σ*_avg_ should be understood as a simulation-derived global response descriptor for comparative ranking within the present model assumptions, rather than as a direct representation of the full final residual-stress field of fabricated components [52,54]. The use of *σ*_avg_ is justified because it is extracted through a fixed volume-averaging rule over the activated deposited region, which reduces sensitivity to local mesh-dependent stress peaks and transient stress concentrations. However, *σ*_avg_ cannot describe directional stress components, local tensile hot spots, or through-thickness stress gradients; it is therefore used only as a stable scalar indicator for process ranking and trend-level comparison.

The machine learning results show that BPNN and LSTM are the most suitable models for the present dataset, while BPNN provides the best overall balance between predictive accuracy, stability, and interpretability for a structured static tabular dataset [55,56]. The machine learning methods used in this work are conventional supervised regression models; the principal contribution is the finite-element-generated *σ*_avg_ dataset and its interpretation using SHAP and PDP analyses. SHAP and PDP analyses consistently indicate that laser power is the dominant control variable in the investigated process window, whereas substrate preheating exerts a secondary mitigating effect, and scanning speed mainly contributes through coupled interactions [33]. Within the present parameter range, the lower predicted *σ*_avg_ is obtained in the intermediate-to-high-power region centered near 180–200 W. This favorable condition should be interpreted as a local optimum within the investigated process space, not as a universal optimum for all geometries, materials, or SLM systems [57].

BPNN was selected as the representative surrogate model because it achieved high predictive accuracy, and the present dataset consists of static tabular process variables rather than sequential data. Thus, its feed-forward structure is more appropriate for this task. BPNN provides comparable accuracy with a simpler feed-forward architecture and is more suitable for SHAP- and PDP-based feature interpretation. Nevertheless, BPNN is not physics-constrained, lacks intrinsic uncertainty quantification, may inherit bias from the simplified finite element model, and should mainly be used for interpolation within the investigated simulation domain.

The experimental observations provide limited but meaningful support for the proposed framework. Metallographic results indicate that lower laser powers are associated with poorer forming quality, whereas samples fabricated near 200 W show a denser morphology [58]. At the macroscopic level, the XRD-derived stress estimates show a variation trend similar to the simulated *σ*_avg_ values [59]. Therefore, the experiments support the practical usefulness of the framework for process-window screening with respect to the dominant variables LP and SS, but they do not establish strict quantitative equivalence between the simulated *σ*_avg_ descriptor and experimentally measured residual stress [53]. The agreement should be interpreted as trend-level consistency rather than validation of the complete residual-stress distribution.

Several limitations should be explicitly acknowledged. First, the finite element model is based on simplified assumptions and does not fully resolve plasticity, stress relaxation, or melt-pool fluid dynamics. Second, the machine learning model was trained on simulation-generated data and should therefore be interpreted within the domain of the simulation assumptions and parameter range. Thus, any systematic bias in the finite element model may be inherited by the surrogate model. Third, the experimental study focused mainly on the two dominant factors identified by the model, namely laser power and scanning speed, while heat-source diameter and substrate preheating temperature were kept constant. Therefore, the effects of HSD and SPH predicted by the surrogate model were not independently validated experimentally, which limits the experimental confirmation of the full four-factor *σ*_avg_-based framework. Accordingly, the proposed framework is best understood as an efficient comparative screening tool for *σ*_avg_-based process evaluation, rather than as a full replacement for high-fidelity thermo-elasto-plastic simulation or exhaustive experimental characterization. Future work should incorporate broader experimental coverage, repeat measurements with statistical analysis, clearer reporting of the *σ*_avg_ extraction procedure and data-screening criteria, and more physics-complete simulation models [60,61]. Further comparison with high-fidelity residual-stress simulations, uncertainty-aware surrogate modeling, and external validation on different geometries or SLM systems would improve the rigor and transferability of the proposed framework.

## 5. Conclusions

In this study, a finite element simulation-driven machine learning surrogate framework was developed for trend-oriented rapid comparative process-window screening in SLM-fabricated 316L stainless steel. Under the assumptions of the present finite element model, the simulated average thermal stress, *σ*_avg_, was used as the primary prediction target and comparative screening indicator. The main conclusions are as follows:

(1) The finite element results indicate that *σ*_avg_, as a volume-averaged simulation-derived stress descriptor, can provide a stable scalar indicator for comparing the overall thermo-mechanical response of different process-parameter combinations. Because *σ*_avg_ is derived from von Mises equivalent stress, it does not represent the full residual-stress tensor field and should not be interpreted as a direct substitute for experimentally measured residual stress.
(2) Among the nine evaluated regression algorithms, BPNN exhibited the best predictive performance for the finite-element-generated tabular dataset. Considering predictive accuracy, stability, and interpretability for the present static tabular problem, BPNN was selected as the representative surrogate model.
(3) Within the investigated process window, the *σ*_avg_-based response showed a clear nonlinear dependence on process parameters. A relatively lower predicted average stress level was observed in the intermediate-to-high-power region centered near 180–200 W, indicating a more favorable thermal–mechanical balance under the present simulation assumptions.
(4) BPNN-based SHAP and PDP analyses revealed that laser power is the dominant governing parameter for the predicted *σ*_avg_ response, whereas substrate preheating temperature plays a secondary mitigating role and scanning speed and heat-source diameter mainly influence the response through secondary or coupled effects.
(5) Limited experimental observations provided trend-level support for the simulation-guided screening results. Metallographic examination showed improved forming quality near 200 W, and the XRD-derived macroscopic stress estimates exhibited a variation trend similar to the simulated *σ*_avg_ values under the tested LP–SS conditions.

Overall, the proposed framework can serve as an efficient tool for comparative process screening within the specific investigated parameter range. However, it should be regarded as a surrogate-based ranking approach built on simplified simulation assumptions and limited experimental trend support, rather than as a direct predictor of the full final residual-stress field of fabricated components. Its applicability should not be arbitrarily extended beyond the current process window and material system.

## Figures and Tables

**Table 1:** Chemical composition of 316L stainless steel. Data are from the work of Chen, X., as reported in ref. [26].

**Element**	**C**	**Mn**	**Si**	**S**	**P**
Content (%)	0.0094	0.051	0.56	0.02	<0.01
**Element**	**Cr**	**Ni**	**Mo**	**O**	**Fe**
Content (%)	17.94	11.92	2.46	0.015	Bal

**Table 2:** Thermal and mechanical properties parameters of 316L stainless steel. Data are from the work of Pichler, P., as reported in ref. [27].

T/K	HS/kJ·kg^−1^	ρIG/μΩ m	ρcorr/μΩ m	D(T)/kg·m^−3^	$\frac{V(T)}{V_{0}}/1$
500	109	0.954	0.970	7770	1.017
600	163	1.007	1.033	7714	1.025
700	218	1.054	1.089	7657	1.033
800	274	1.093	1.138	7601	1.041
900	333	1.128	1.183	7544	1.049
1000	393	1.157	1.223	7488	1.057
1100	454	1.182	1.259	7432	1.065
1200	515	1.203	1.290	7375	1.073
1300	554	1.218	1.316	7319	1.081
1400	626	1.232	1.342	7263	1.089
1500	697	1.247	1.368	7206	1.097
1600	769	1.262	1.394	7150	1.105
1700	1042	1.291	1.464	6964	1.132
1800	1190	1.299	1.495	6857	1.151
1900	1275	1.301	1.514	6791	1.163
2000	1360	1.303	1.532	6725	1.176
2100	1444	1.305	1.551	6659	1.189
2200	1529	1.307	1.570	6593	1.201
2300	1614	1.309	1.589	6527	1.214
2400	1699	1.311	1.608	6461	1.226
2500	1783	1.313	1.627	6395	1.239
2600	1868	1.315	1.646	6329	1.251
2700	1953	1.317	1.665	6262	1.264
2800	2037	1.319	1.684	6196	1.277

**Table 3:** Selection of laser melting test design.

Test Number	Laser Power (W)	Scanning Speed (mm/s)	Test Number	Laser Power (W)	Scanning Speed (mm/s)
1	120	950	8	120	1050
2	140	950	9	140	1050
3	160	950	10	160	1050
4	180	950	11	180	1050
5	200	950	12	200	1050
6	220	950	13	220	1050
7	240	950	14	240	1050

**Table 4:** Summary of predictive errors for the nine regression models.

Error in the Dataset		Machine Learning Algorithms								
		MLR	LSBoost	GPR	GKR	GAM	DTR	RF	BPNN	LSTM
Training set	MAE	8.985	1.265	1.468	1.665	3.402	3.974	1.085	1.038	1.101
	MAPE	0.055	0.008	0.010	0.011	0.022	0.025	0.007	0.007	0.007
	MSE	136.2	2.7	3.8	5.8	18.6	27.6	2.2	2.3	2.5
	RMSE	11.668	1.643	1.953	2.402	4.311	5.258	1.481	1.500	1.575
	R^2^	0.852	0.997	0.996	0.994	0.980	0.970	0.998	0.998	0.997
Test set	MAE	9.903	3.153	2.088	2.403	4.843	5.693	3.106	1.318	1.569
	MAPE	0.063	0.021	0.014	0.016	0.031	0.037	0.020	0.009	0.011
	MSE	140.0	14.3	7.7	10.7	37.6	56.7	16.9	3.2	3.9
	RMSE	11.831	3.780	2.772	3.265	6.136	7.528	4.113	1.801	1.987
	R^2^	0.886	0.988	0.994	0.991	0.969	0.953	0.986	0.997	0.997
Validation set	MAE	9.848	2.699	2.040	2.072	3.894	6.786	3.421	1.075	1.169
	MAPE	0.060	0.018	0.014	0.014	0.024	0.043	0.022	0.007	0.008
	MSE	192.7	13.1	7.6	7.8	29.3	72.3	21.2	2.0	3.2
	RMSE	13.881	3.616	2.758	2.797	5.410	8.503	4.607	1.407	1.783
	R^2^	0.792	0.987	0.993	0.991	0.967	0.918	0.976	0.998	0.996
Five-fold cross-validation	Fold 1	8.99	2.58	1.79	1.95	4.30	6.68	4.23	1.48	1.10
	Fold 2	9.40	2.70	2.10	2.20	4.20	5.50	4.40	1.70	1.20
	Fold 3	9.11	2.76	2.15	2.92	3.69	6.67	4.47	1.34	1.19
	Fold 4	9.09	2.77	1.91	2.51	4.22	6.05	3.79	1.84	1.25
	Fold 5	10.00	2.70	1.90	2.20	4.10	6.70	4.20	1.60	1.30

**Table 5:** XRD γ(220) peak shift and corresponding macroscopic stress estimates of SLM-fabricated 316L stainless steel under different process parameters.

Scanning Speed (mm/s)	Laser Power (W)	Mean 2*θ* Meas. (°)	Mean Δ2*θ* (°)	SD of Δ2*θ* (°)	Mean Stress *σ* (MPa)	Stress SD (MPa)
950	120	74.518	−0.179	0.007	203.78	5.85
	140	74.557	−0.140	0.005	157.16	4.15
	160	74.567	−0.130	0.007	145.72	5.85
	180	74.586	−0.111	0.005	123.73	4.33
	200	74.623	−0.074	0.004	82.68	3.51
	220	74.615	−0.082	0.005	93.82	4.42
	240	74.581	−0.116	0.002	130.77	1.34
1050	120	74.513	−0.184	0.010	205.54	8.85
	140	74.549	−0.148	0.004	166.84	3.56
	160	74.580	−0.117	0.014	128.13	12.14
	180	74.617	−0.080	0.012	87.08	10.14
	200	74.630	−0.067	0.017	70.96	14.99
	220	74.609	−0.088	0.012	95.88	10.81
	240	74.566	−0.131	0.010	145.14	8.39

**Figure A1:**
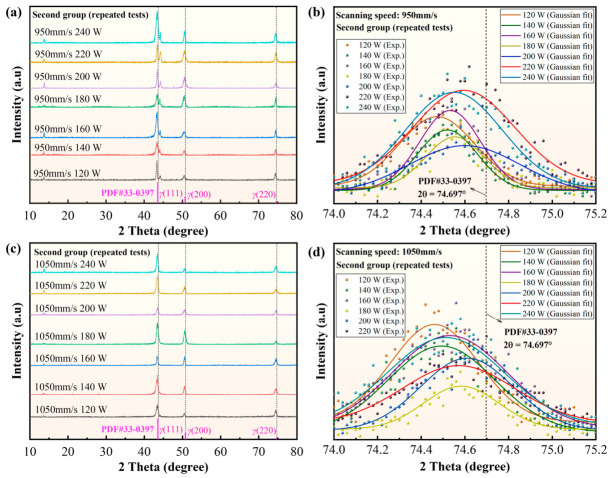
XRD results and Gaussian fitting of the γ(220) crystal plane for SLM-fabricated 316L stainless steel under different process parameters (repeated experiment Set 2). (**a**) XRD patterns at 950 mm/s under different powers; (**b**) Gaussian fitting of the 74–75.2° peak at 950 mm/s; (**c**) XRD patterns at 1050 mm/s under different powers; (**d**) Gaussian fitting of the 74–75.2° peak at 1050 mm/s.

**Figure A2:**
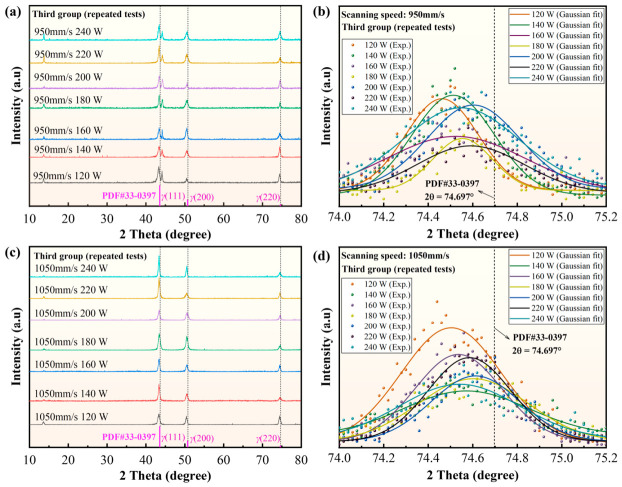
XRD results and Gaussian fitting of the γ(220) crystal plane for SLM-fabricated 316L stainless steel under different process parameters (repeated experiment Set 3). (**a**) XRD patterns at 950 mm/s under different powers; (**b**) Gaussian fitting of the 74–75.2° peak at 950 mm/s; (**c**) XRD patterns at 1050 mm/s under different powers; (**d**) Gaussian fitting of the 74–75.2° peak at 1050 mm/s.

## Data Availability

The original contributions presented in this study are included in the article. Further inquiries can be directed to the corresponding author.

## Abbreviations

The following abbreviations are used in this manuscript:

AM	Additive Manufacturing
SLM	Selective Laser Melting
ML	Machine Learning
XRD	X-Ray Diffraction
MLR	Multiple Linear Regression
DTR	Decision Tree Regression
GPR	Gaussian Process Regression
RF	Random Forest Regression
LSTM	Long Short-Term Memory
BPNN	Backpropagation Neural Network
MAE	Mean Absolute Error
MAPE	Mean Absolute Percentage Error
MSE	Mean Squared Error
RMSE	Root Mean Squared Error
LP	Laser Power
SS	Scanning Speed
HSD	Heat-Source Diameter
SPH	Substrate Preheating Temperature
PDP	Partial Dependence Plot
SHAP	Shapley Additive Explanations
SD	Standard Deviation

## Appendix A

### Moving Gaussian Heat-Source Subroutine

The APDL code listed below is provided only to illustrate the implementation logic of the moving Gaussian heat source for one representative simulation case. Because the full dataset used in this study was generated through parameterized batch simulations with different LP, SS, HSD, and SPH settings, the code shown here should be regarded as an example of the heat-source implementation rather than the complete parameter-sweeping script used for all simulations.

*DEL,_FNCNAME

*DEL,_FNCMTID

*DEL,_FNCCSYS

*SET,_FNCNAME,’cccc’

*SET,_FNCCSYS,0

! /INPUT,.\func\N0004.func,,,1

*DIM,%_FNCNAME%,TABLE,6,38,1,,,,%_FNCCSYS%

!

! Begin of equation: 2*(200)/(3.1415926*(0.00005)^2)*exp(−2*((−0.001 + 1*

! (−0.013))^2 + (−0.0003)^2)/(0.00005)^2)*0.5

*SET,%_FNCNAME%(0,0,1), 0.0, −999

*SET,%_FNCNAME%(2,0,1), 0.0

*SET,%_FNCNAME%(3,0,1), 0.0

*SET,%_FNCNAME%(4,0,1), 0.0

*SET,%_FNCNAME%(5,0,1), 0.0

*SET,%_FNCNAME%(6,0,1), 0.0

*SET,%_FNCNAME%(0,1,1), 1.0, −1, 0, 2, 0, 0, 0

*SET,%_FNCNAME%(0,2,1), 0.0, −2, 0, 200, 0, 0, −1

*SET,%_FNCNAME%(0,3,1), 0, −3, 0, 1, −1, 3, −2

*SET,%_FNCNAME%(0,4,1), 0.0, −1, 0, 0.00004, 0, 0, 0

*SET,%_FNCNAME%(0,5,1), 0.0, −2, 0, 2, 0, 0, −1

*SET,%_FNCNAME%(0,6,1), 0.0, −4, 0, 1, −1, 17, −2

*SET,%_FNCNAME%(0,7,1), 0.0, −1, 0, 3.1415926, 0, 0, −4

*SET,%_FNCNAME%(0,8,1), 0.0, −2, 0, 1, −1, 3, −4

*SET,%_FNCNAME%(0,9,1), 0.0, −1, 0, 1, −3, 4, −2

*SET,%_FNCNAME%(0,10,1), 0.0, −2, 0, 0, 0, 0, 0

*SET,%_FNCNAME%(0,11,1), 0.0, −3, 0, 1, 0, 0, −2

*SET,%_FNCNAME%(0,12,1), 0.0, −4, 0, 1, −2, 2, −3

*SET,%_FNCNAME%(0,13,1), 0.0, −2, 0, 2, 0, 0, −4

*SET,%_FNCNAME%(0,14,1), 0.0, −3, 0, 1, −4, 3, −2

*SET,%_FNCNAME%(0,15,1), 0.0, −2, 0, 0.001, 0, 0, 2

*SET,%_FNCNAME%(0,16,1), 0.0, −4, 0, 1, 2, 2, −2

*SET,%_FNCNAME%(0,17,1), 0.0, −2, 0, 0.013684, 0, 0, 1

*SET,%_FNCNAME%(0,18,1), 0.0, −5, 0, 1, 1, 2, −2

*SET,%_FNCNAME%(0,19,1), 0.0, −2, 0, 0.95, 0, 0, −5

*SET,%_FNCNAME%(0,20,1), 0.0, −6, 0, 1, −2, 3, −5

*SET,%_FNCNAME%(0,21,1), 0.0, −2, 0, 1, −4, 1, −6

*SET,%_FNCNAME%(0,22,1), 0.0, −4, 0, 2, 0, 0, −2

*SET,%_FNCNAME%(0,23,1), 0.0, −5, 0, 1, −2, 17, −4

*SET,%_FNCNAME%(0,24,1), 0.0, −2, 0, 0.0003, 0, 0, 3

*SET,%_FNCNAME%(0,25,1), 0.0, −4, 0, 1, 3, 2, −2

*SET,%_FNCNAME%(0,26,1), 0.0, −2, 0, 2, 0, 0, −4

*SET,%_FNCNAME%(0,27,1), 0.0, −6, 0, 1, −4, 17, −2

*SET,%_FNCNAME%(0,28,1), 0.0, −2, 0, 1, −5, 1, −6

*SET,%_FNCNAME%(0,29,1), 0.0, −4, 0, 1, −3, 3, −2

*SET,%_FNCNAME%(0,30,1), 0.0, −2, 0, 0.00004, 0, 0, 0

*SET,%_FNCNAME%(0,31,1), 0.0, −3, 0, 2, 0, 0, −2

*SET,%_FNCNAME%(0,32,1), 0.0, −5, 0, 1, −2, 17, −3

*SET,%_FNCNAME%(0,33,1), 0.0, −2, 0, 1, −4, 4, −5

*SET,%_FNCNAME%(0,34,1), 0.0, −2, 7, 1, −2, 0, 0

*SET,%_FNCNAME%(0,35,1), 0.0, −3, 0, 1, −1, 3, −2

*SET,%_FNCNAME%(0,36,1), 0.0, −1, 0, 0.5, 0, 0, −3

*SET,%_FNCNAME%(0,37,1), 0.0, −2, 0, 1, −3, 3, −1

*SET,%_FNCNAME%(0,38,1), 0.0, 99, 0, 1, −2, 0, 0

! End of equation: 2*(200)/(3.1415926*(0.00005)^2)*exp(−2*((−0.0009 + 1*

! (−0.003))^2 + (−0.0003)^2)/(0.00005)^2)*0.5

!-->

SF,A14,HFLUX,%cccc%

## Appendix B

This appendix provides supplementary repeated XRD measurements for SLM-processed 316L stainless steel. Figure A1 and Figure A2 show the XRD patterns and Gaussian fitting of the γ(220) crystal plane from repeated experiments. Table A1 lists three sets of raw data for diffraction offset Δ2*θ* and residual stress under different process parameters, verifying experimental repeatability.

**Table A1:** Three repeated XRD measurements of Δ2*θ* and residual stress under different SLM process parameters.

Scanning Speed (mm/s)	Laser Power (W)	Δ2*θ* (°)			Residual Stress *σ* (MPa)		
		1st Test	2nd Test	3rd Test	1st Test	2nd Test	3rd Test
950	120	−0.236	−0.224	−0.235	207.594	197.038	206.714
	140	−0.175	−0.177	−0.184	153.936	155.696	161.853
	160	−0.160	−0.164	−0.173	140.742	144.260	152.177
	180	−0.135	−0.143	−0.144	118.751	125.788	126.668
	200	−0.090	−0.098	−0.094	79.167	86.204	82.686
	220	−0.112	−0.102	−0.106	98.519	89.723	93.241
	240	−0.149	−0.147	−0.150	131.066	129.306	131.945
1050	120	−0.243	−0.223	−0.235	196.159	206.714	213.752
	140	−0.186	−0.189	−0.194	166.251	170.649	163.612
	160	−0.151	−0.130	−0.156	114.353	137.223	132.825
	180	−0.103	−0.086	−0.108	75.649	95.001	90.603
	200	−0.082	−0.063	−0.097	55.417	85.325	72.130
	220	−0.114	−0.095	−0.118	83.565	103.797	100.278
	240	−0.166	−0.155	−0.174	136.344	153.057	146.02
